# UCHL1 Regulates Lipid and Perilipin 2 Level in Skeletal Muscle

**DOI:** 10.3389/fphys.2022.855193

**Published:** 2022-04-07

**Authors:** Ryan Antony, Katherine Aby, Hongbo Gao, Mary Eichholz, Rekha Srinivasan, Yifan Li

**Affiliations:** Division of Basic Biomedical Sciences, University of South Dakota Sanford School of Medicine, Vermillion, SD, United States

**Keywords:** skeletal muscle, lipid, ubiquitin C-terminal hydrolase L1, perilipin 2, mice, C2C12 cell

## Abstract

Ubiquitin C-terminal hydrolase L1 (UCHL1) is a deubiquitinating enzyme that was originally found in neurons. We found that UCHL1 is highly expressed in slow oxidative skeletal muscles, but its functions remain to be fully understood. In this study, we observed that UCHL1 protein levels in skeletal muscle and C2C12 myotubes were downregulated by fasting or glucose starvation respectively. Skeletal muscle selective knockout (smKO) of UCHL1 resulted in a significant reduction of lipid content in skeletal muscle and improved glucose tolerance. UCHL1 smKO did not significantly change the levels of key proteins involved in oxidative metabolism such as SDHA, Akt, or PDH. Interestingly, while the levels of the major lipases and lipid transporters were unchanged, perilipin 2 was significantly downregulated in UCHL1 smKO muscle. Consistently, in C2C12 myotubes, UCHL1 siRNA knockdown also reduced perilipin 2 protein level. This data suggests that UCHL1 may stabilize perilipin 2 and thus lipid storage in skeletal muscle.

## Introduction

Skeletal muscle is the largest tissue in the body and is critical for metabolism. Skeletal muscles are highly heterogenous and plastic in terms of metabolism and contractility. Based on the nature of their metabolism and contractile activities, skeletal muscle fibers are roughly classified as slow oxidative (type I), fast oxidative (type IIa and IIx), and fast glycolytic (type IIb) fibers (Schiaffino and Reggiani, 2011). The oxidative fibers can use both glucose and fatty acids as energy fuels to generate ATP. The ability of muscle fibers to shift fuel preferences due to various factors and stimuli is known as metabolic plasticity (Kelley, 2005). Skeletal muscle metabolic plasticity is attenuated or diminished in obesity, diabetes, and aging (Storlien et al., 2004), suggesting that metabolic plasticity is critical for whole body metabolic homeostasis.

Fatty acids oxidation (FAO) is the major energy source for oxidative skeletal muscles (Hirabara et al., 2007; Silveira et al., 2008; Turner et al., 2014; Lundsgaard et al., 2018). Free fatty acids (FFAs) are taken up into cells via specific fatty acid transport proteins such as CD36 and fatty acid binding protein (FABP). In the cytosol, FFAs are converted into acyl-coenzyme A (CoA), which is the substrate for FAO as well as lipid synthesis. FAO is carried out in mitochondria through a cyclic process of a series of enzymatic reactions. Muscle lipid homeostasis is determined by fatty acid uptake, β-oxidation, lipid synthesis, and lipolysis (Kelley, 2005; Houten et al., 2016). Intramuscular lipid content is increased with excess fatty acids availability or reduced fatty acid oxidation (Dyck et al., 1997). Impaired fatty acid metabolism and increases intramuscular lipid accumulation in obesity are linked to muscle inflammation (Das, 2001; Reidy et al., 2018) and insulin resistance (Stein and Wade, 2005; Koves et al., 2008; Turcotte and Fisher, 2008; Samuel et al., 2010; Dirks et al., 2016; Dominguez and Barbagallo, 2016; Lalia et al., 2016). On the other hand, endurance exercise training also increases intramuscular lipid, which does not cause insulin resistance but rather increases insulin sensitivity. This phenomenon is known as the athletic paradox (Goodpaster et al., 2001; Dubé et al., 2008). These facts suggest that it is not the static intramuscular lipid content but the lipid mobilization of the stored lipid that is critical for muscle metabolism and insulin sensitivity. The regulation of intramuscular lipid storage and utilization remain to be fully understood.

The excess FFA in the cytosol will be converted to triglyceride (TG) and stored in lipid droplets (LDs) in the cells (Wang, 2016; Ogasawara et al., 2020) as intramuscular lipids. LDs are the dynamic organelles that control lipid synthesis, storage, mobilization, and lipolysis (Walther and Farese, 2012; Olzmann and Carvalho, 2019). LDs contain neutral lipid enveloped with a phospholipid monolayer embedded with several proteins. The most abundant LD associated proteins are perilipins (Kimmel et al., 2010). Of five members of perilipin family, perilipin 2, 3, and 5 are expressed in skeletal muscle and perilipin 2 is best characterized for its function in regulation of LDs and muscle lipid content (Conte et al., 2016). Perilipin 2 is positively correlated with muscle lipid content (Minnaard et al., 2009). Perilipin 2 protein can be degraded through chaperone-mediated autophagy pathway (Kaushik and Cuervo, 2015) or ubiquitin-proteasome pathway (Xu et al., 2005; Masuda et al., 2006).

Ubiquitin C-terminal hydrolase L1 (UCHL1) is highly expressed in the nervous system and functions as a deubiquitinating enzyme. UCHL1 is also expressed in some peripheral tissues, including pancreas, liver, some cancer tissues, as well as skeletal muscles, yet its function in skeletal muscle needs to be better understood. Our recent work showed that skeletal muscle UCHL1 is involved in mTORC1 activity (Gao et al., 2019). In this study, we report that muscle UCHL1 affects intramuscular lipid metabolism by stabilizing perilipin 2.

## Methods

### Animals

All experimental protocols and use of animals in this study were reviewed and approved by the University of South Dakota Institutional Animal Care and Use Committee (IACUC) and followed the NIH guideline of animal use in research under protocol No. 1-03-19-22D. As previously mentioned (Gao et al., 2020), the mouse line carrying floxed UCHL1 was generated from a strain of “UCHL1 HEPD0603_7_h04” provided by the UK Medical Research Council on behalf of the European Mouse Mutant Archive (EMMA). The mouse strain with skeletal muscle specific knockout (smKO) of UCHL1 was generated by crossing a mouse carrying floxed UCHL1 and a mouse expressing cre under the skeletal muscle specific myosin light polypeptide promoter (The Jackson Laboratory, stock # 024713). For genotyping as well as for identification, a singular toe was taken from each new born mouse around 5 days after birth. The genome DNA was extracted from the toe tissue using protease K digestion method. The genotype of each mouse was confirmed using PCR with primers for floxed UCHL1 and Cre.

### Glucose Tolerance and Insulin Tolerance Tests

Glucose tolerance testing (GTT) and insulin tolerance test (ITT) were done in 3-month-old WT and UCHL1 smKO mice. Both groups of mice were trained for several days before testing by placing the mice in a 50 ml tube for 2 minutes and allowing them to adapt to the testing environment. Food was removed from cages the night prior to testing to allow for a ~12 h fasting period. Mice were placed in the tube for 1 min, a cut was made at the end of the tail and the blood glucose was measured using a OneTouch Ultra 2 blood glucose meter (LifeScan, Pennsylvania, United States). Blood glucose was taken before (baseline) and at 15, 30, 60, 90, and 120 min after intraperitoneal injection of glucose solution (2 g/kg) or insulin saline (2 U/kg). The glucose tolerance curves were generated and the areas under curve (AUC) were analyzed using GraphPad Prism 9.0.

### Tissue Collection

As previously described (Gao et al., 2020), mice were anesthetized using isoflurane (3–4%). Muscles of the hind limb were exposed by removing the skin. The soleus (slow oxidative fibers) and extensor digitorum longus (EDL) (fast glycolytic fibers) muscles were collected in DNase/RNase free tubes and frozen on dry ice for Western blot assay. For tissue staining, soleus muscles were isolated and snap frozen in pre-chilled 2-methylbutane (Kumar et al., 2015), then embedded in optimal cutting temperature compound (OCT) on dry ice and stored at −70°C for future cryosectioning. Muscles were sectioned into 10-15 µm sections and adhered onto slides for staining.

### Muscle Staining

Intramuscular lipid was stained using Bodipy or oil red O staining.

Bodipy staining was based on published works (Spangenburg et al., 20112011; Qiu and Simon, 2016) with minor modifications. Slides were removed from the −70°C freezer and immediately fixed with 4% paraformaldehyde (PFA) for 15 min. Slides were then washed in PBS 3 times for 5 min each, and then incubated for 30 min at room temperature in a Bodipy 493/503 (ThermoFisher, D3922) in DMSO with a concentration of 3.8 mM. Following 3 washes in PBS for 5 min each, slides were mounted with Fluoromount-G solution (Southern Biotech, 0100-01) for imaging.

The oil red o staining was performed using the oil red o kit (VitroVivo Biotech, VB-3007) following the manufacturer’s protocol with modifications. Slides were brought to room temperature for 30 min and fixed in 10% formalin for 20 min, followed by 30 min of air drying at room temperature. Slides were submerged in the pre-stain solution for 5 min, then incubated in pre-warmed Oil Red O solution at 60°C for 10 min, and immediately submerged into pre-warmed differentiating solution for 5 min at 60°C. The slides were removed from the solution and rinsed in 2 changes of Milli Q pure water before submerging in Myers Hematoxylin solution for 20–30 s at room temperature. The slides were then washed with tap water for 3 min, rinsed with Milli Q pure water, then mounted with Fluoromount-G solution (Southern Biotech, 0100-01).

For fluorescent staining for type I muscle fiber and UCHL1, muscle sections were fixed with 4% paraformaldehyde, washed with PBST, and incubated overnight with mouse antibody for type I myosin heavy chain (Developmental Studies Hydridoma Bank, BA-D5) and rabbit antibody for UCHL1 (Abcam, ab108986). Following 3 washes with PBST, the sections were incubated with secondary antibodies goat-anti-mouse conjugated with Alexa-488 and goat-anti-rabbit conjugated with Alexa-594 (Invitrogen). Following 3 washes with PBST, the sections were mounted with Fluoromount-G and imaged using a fluorescent microscope (Olympus).

### Cell Culture and Gene Knockdown

As previously described (Antony and Li, 2020), C2C12 myoblasts were cultured in complete media (CM) made of dulbecco’s modified eagle’s medium (DMEM, ThermoFisher-Gibco) containing 10% fetal bovine serum (FBS), 1% penicillin-streptomycin, and 1% HEPES. For glucose and serum starvation experiments, fully confluent cells were switched into fresh CM, incomplete media (ICM, FBS free), or FBS-free and glucose-free media (NG) overnight and then harvested for Western blot. To achieve UCHL1 knockdown (KD), once fully confluent the cells were switched into 1 ml ICM and treated with a mixture of Lipofectamine RNAiMAX (ThermoFisher, 13,778,075) and UCHL1 siRNA (IDT) for approximately 8 h. Following this, 1 ml of differentiating media (DM) made of DMEM containing 2% horse serum, 1% penicillin-streptomycin, and 1% HEPES was added to the cells. Following the overnight incubation, cells were switched to 2 ml of fresh DM. DM was changed every 72 h for a total incubation time of 12 days before being harvested for Western blot (WB).

### Total Protein Extraction and Western Blot

Soleus muscle tissues were homogenized in 1X RIPA buffer containing 1% protease inhibitor cocktail (Research Products International, P50600-1), 1% phosphatase inhibitor cocktail (Research Products International, P52104-1), 0.1% SDS, and 0.1% MG132. The muscle was placed in a 1.5 ml tube and homogenized using a plastic pestles connected to an electric driver. The tissue was crushed prior to adding the above buffer, and then continuously homogenized for about 30 s in the buffer. The homogenates were allowed to set in the buffer for approximately 30 min on ice before being homogenized one more time. Tubes were then spun down at 10,000 xg for 5 min at 4°C. Protein concentration of the supernatants of muscle homogenates was determined by a standard BCA assay. The protein concentration of all samples were normalized to the same concentration. Cells were homogenized using the same buffer and concentrations of cocktails.

Western blot was performed as described previously (Gao et al., 2020). In addition to a mass ladder (BioRad Precision Plus Protein All Blue Standard) loaded into each end lane, 15 µl of homogenized muscle/cell samples containing loading buffer at a concentration of approximately 2.5 μg/μl were subject to electrophoresis in 11–16% gradient gels at 100 V for approximately 3 h. Proteins were transferred on to 0.22 µM nitrocellulose membranes at 350 mA using a trans-blot apparatus (Bio-Rad, Hercules, CA). The membranes were fixed in 50% methanol for 30 min at 4°C followed by 30 min at 37°C. The membranes were then blocked with 3% non-fat milk in PBST for 1 h on a rocker at room temperature. The membranes were then incubated with primary antibodies and 0.5% BSA in PBST overnight at 4°C. The following antibodies were used: anti UCHL1 (Abcam, 108986), GAPDH (Santa Cruz, sc-47724), Actin (Santa Cruz, sc-47778), DGAT2 (Santa Cruz, sc-293211), ATGL (Cayman, 10,006,409), Perilipin 2 (Novus, NB110-40877), Perilipin 3 (Novus, NB110-40764), OXPAT (Novus, NB110-60509), MAGL (Cayman, 100035), CD36 (Protein tech, 18836-1-AP), Akt (Cell Signaling Technologies, 9,272), phosphor-Akt (Cell Signaling Technologies, 4,051), AMPK (Cell Signaling Technologies, 2,793), phosphor-AMPK (Cell Signaling Technologies, 2,535), SDHA (Abcam, ab14715), SDHB (Abcam, ab178423), PDH (Cell Signaling Technologies, 3,205), and HSL (Cayman, 10,006,371). Following 3 washes with PBST for 5 min each, membranes were incubated with the appropriate secondary antibodies conjugated with Alexa-680 or 800 (Invitrogen) for 1 h at room temperature followed by 2 washes with PBST and 1 wash with PBS. The protein bands on the membrane were imaged using a LICOR scanner (LICOR Biosciences, Lincoln, NE). Following the imaging, some membranes were stained for total protein load using Imperial Protein Stain solution (ThermoFisher, UF286575) and de-stained with 50% methanol and 10% glacial acetic acid. Band densities of the proteins were analyzed using NIH ImageJ software and normalized against total protein stain bands or GAPDH in tissue samples or Actin in cell samples; the ratio of UCHL1 and other proteins were then calculated and compared between KO/KD and WT/Control.

### Triglyceride Assay

The triglyceride content in muscle homogenates was measured using the triglyceride colorimetric assay kit (Cayman, 10,010,303) by following the manufacturer’s protocol. In a 96 well plate, a standard curve was prepared using the included standard reagents and diluents. In each well, 10 µL of sample and 150 µL of the assay enzyme solution were added, thoroughly mixed on a microplate shaker (FisherBrand, 88,861,023), and then incubated for 30 min at 37°C. The absorbance of theassay was measured using a TECAN plate reader (TECAN, Infinite m200) and Magellan software. Absorbance of samples was analyzed to determine differences between WT and KO groups.

### Data Analysis

All data calculations, descriptive statistics, and graphing were performed using Microsoft Excel and GraphPad Prism 9.0. To quantify Western blot results, a protein band density was normalized by total protein stain, GAPDH or beta actin as loading controls. The mean value of the WT/Control group was calculated, followed by calculating the ratio between each individual sample to that of the WT/Control mean. The mean values of the ratio of WT/Control and KO/KD samples were compared between two groups by the two-tailed *t*-test; Statistical significance was defined as *p* value less than 0.05. Data was presented as mean ± SD. To quantify glucose testing results, the mean values of glucose at each time point and an area under curve for both the WT and KO groups were calculated and compared using a two-tailed *t*-test to determine the statistical significance between the two groups.

## Result

### UCHL1 Level is Downregulated by Fasting

**FIGURE 1:**
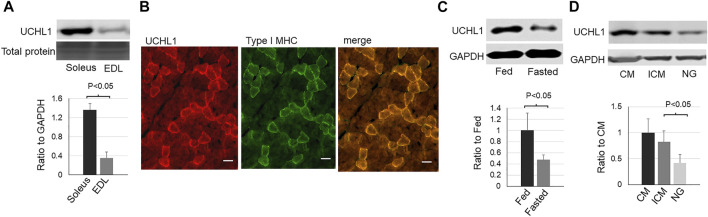
Western blot images (top) and quantifications (bottom) of UCHL1 in mouse skeletal muscles or C2C12 myotubes. **(A)**: UCHL1 protein levels in slow oxidative muscle soleus and fast glycolytic muscle EDL (n = 4); **(B)**: Immunofluorescent staining for UCHL1 (red) and type I myosine heavy chain (green) in soleus muscle section. The scale bar = 20 μm). **(C)**: UCHL1 protein levels in soleus from fed or fasted mice (n = 3 per group); **(D)**: UCHL1 protein levels in C2C12 myotubes complete media (CM, containing 10% FBS and 4.5 g/L glucose), incomplete media (ICM, containing 4.5 g/L glucose but no FBS), or the media without FBS and glucose (NG). (n = 4 per group).

In skeletal muscle, UCHL1 is highly expressed in soleus, a typical slow oxidative muscle, and is very low in EDL, a typical fast glycolytic muscle (Figure 1A). Immunofluorescent staining showed the colocalization of UCHL1 and type I slow oxidative fibers (Figure 1B). Fasting, which promotes muscles fatty acid oxidation, downregulated UCHL1 in soleus (Figure 1C). Consistent with this, in differentiated C2C12 myotubes, UCHL1 level was also downregulated by glucose starvation (Figure 1D). Since fasting is known to shift muscle energy metabolism toward lipid oxidation, this data suggests that UCHL1 may be involved in lipid metabolism in skeletal muscle.

### UCHL1 smKO Reduced Lipid Content in Skeletal Muscle

**FIGURE 2:**
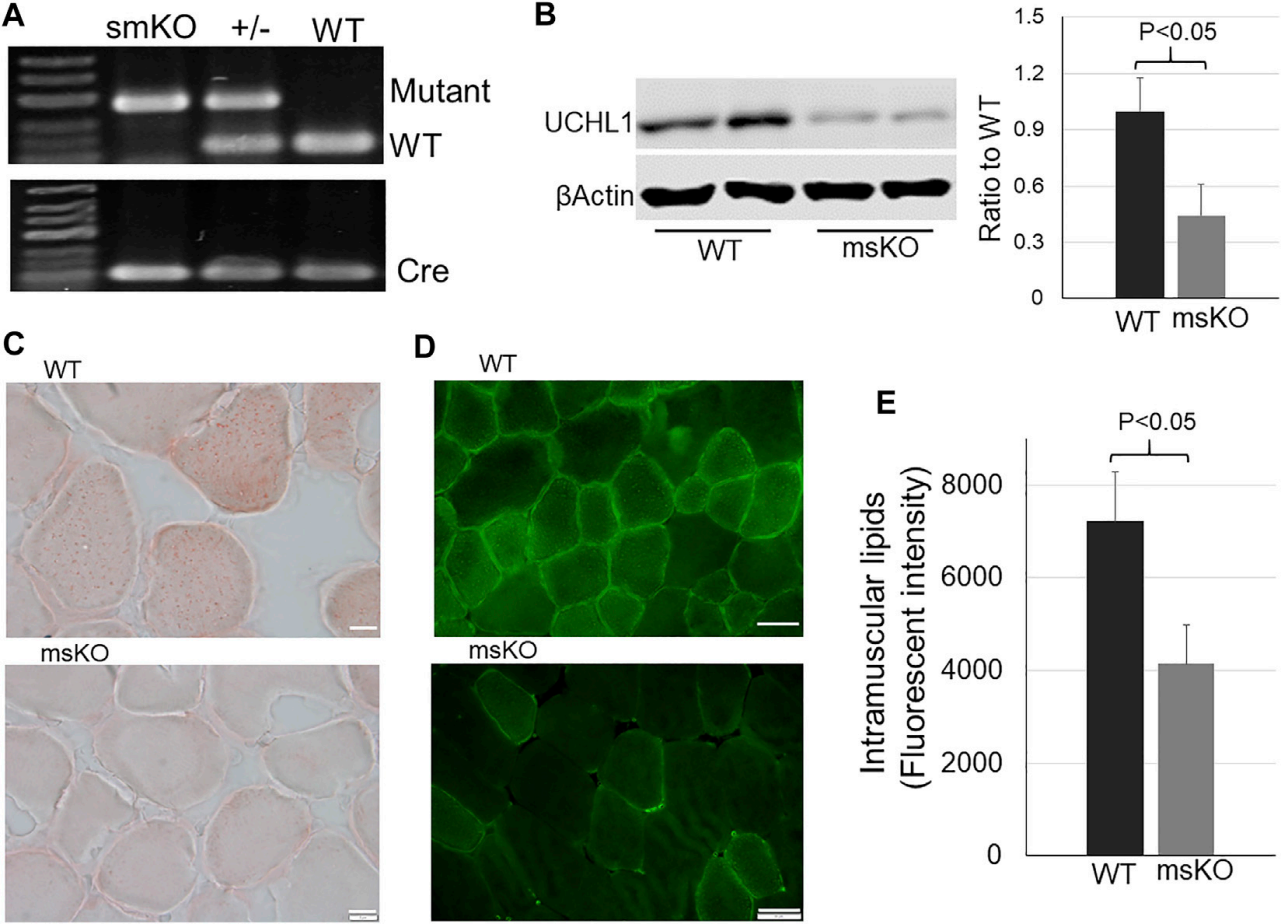
Lipid content in skeletal muscle. **(A)**: Gel images of PCR genotyping to identify smKO, heterozygous, and WT genotypes; **(B)**: Western blot for UCHL1 in muscle from WT and UCHL1 smKO mice. n = 4. **(C)**: Oil-red-O staining for lipid in muscle from WT or UCHL1 smKO mice. The scale bar = 5 μm. **(D)**: Bodipy staining for lipid in muscle from WT or UCHL1 smKO mice. The scale bar = 20 μm. **(E)**: Lipid (triglyceride) in muscle homogenate of WT or UCHL1 smKO mice. n = 4 per group.

To test the functional role of UCHL1 in skeletal muscle, we have generated skeletal muscle specific knockout (smKO) of UCHL1. The genotype of homozygous floxed UCHL1 and cre transgene was confirmed by PCR and a significant reduction of UCHL1 protein level in skeletal muscle was confirmed by Western blot (Figure 2A). The intramuscular lipid content was measured by oil-red-o staining (Figure 2B), BODIPY staining (Figure 2C), and triglyceride assay (Figure 2D). As shown in these panels, the intramuscular lipid content was significantly reduced in the muscle from UCHL1 smKO mice when compared with WT. This data suggests that skeletal muscle UCHL1 may play an essential role in maintaining lipid content in skeletal muscle.

### UCHL1 smKO did Not Affect the Levels of Key Proteins Involved in Metabolism

**FIGURE 3:**
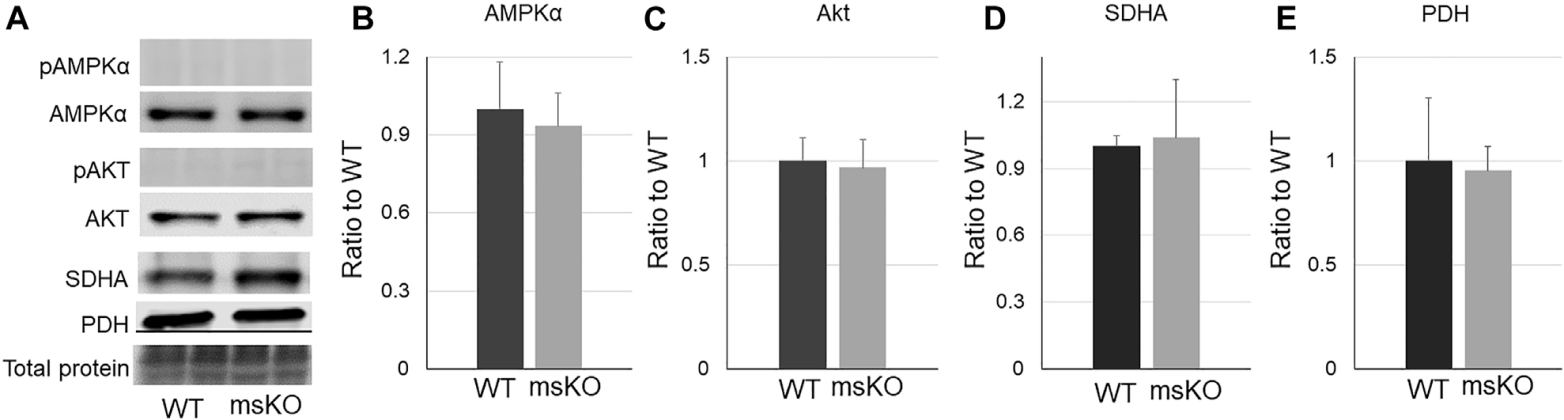
Western blot images **(A)** and quantifications **(B–E)** of muscle samples from WT and UCHL1 smKO mice for key proteins that are involved in lipid metabolisms, including AMPKα (**(B)**, n = 5), Akt (**(C)**, n = 3), SDHA (**(D)**, n = 5), and PHD (**(E)**, n = 3).

Intramuscular lipid content can be affected by overall metabolism. We then measured the level of some key proteins that regulate metabolism and mitochondrial function, including phosphorylated and total AMPKα (Figure 3B), phosphorylated and total Akt (C), succinate dehydrogenase (SDH) (Figure 3D), and pyruvate dehydrogenase (PDH) (Figure 3E). However, none of these proteins were altered in the UCHL1 smKO muscle. The levels of phosphorylated Akt and AMPK were nearly undetectable in both WT and UCHL1 smKO samples.

### Reduced perilipin2 in UCHL1 smKO Muscle

**FIGURE 4:**
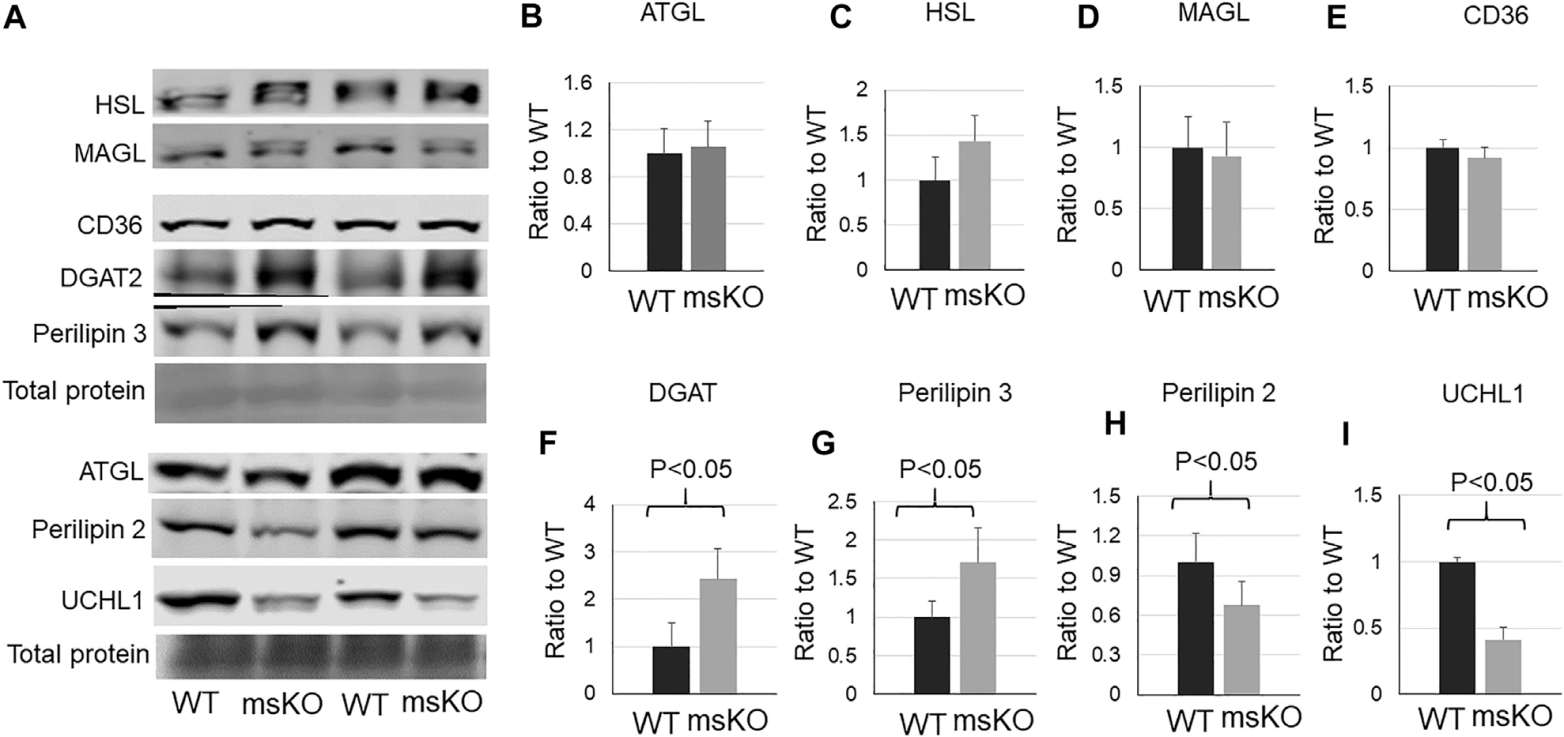
Western blot images **(A)** and quantifications **(B–I)** of muscle samples from WT and UCHL1 smKO mice for lipase ATGL **(B)**, HSL **(C)**, and MAGL **(D)**, the fatty acid transport protein CD36 **(E)**, the key lipid synthesis enzyme DGAT2 **(F)**, perilipin 3 **(G)**, perilipin 2 **(H)**, and UCHL1 **(I)**. n = 4-5 per group.

Lipid content is determined by fatty acid transport, lipid synthesis, lipolysis, and lipid storage. We then assessed the level of proteins that are related to these functions. CD36, which is the major protein responsible for fatty acid transport, was not altered in UCHL1 smKO muscle (Figure 4E). The levels of three major lipases, ATGL (Figure 4B), HSL (Figure 4C), and MAGL (Figure 4D), also remained unchanged in UCHL1 smKO muscle, suggesting that the reduction of lipid content in UCHL1 smKO muscle is unlikely due to the increase in lipolysis activity. Interestingly, perilipin2, a key protein that is associated with and stabilize lipid droplets, was significantly reduced in UCHL1 smKO muscle (Figure 4H), suggesting the possibility that UCHL1 may be essential to stabilize perilipin 2 and thus lipid storage. Perilipin 3 level (Figure 4G) was significantly upregulated in UCHL1 smKO muscle, potentially a compensatory response to the reduction of perilipin2. The major lipid synthase DGAT2 was also upregulated in UCHL1 smKO, which may also be a compensatory response to the reduced lipid content.

### Perilipin 2 Was Downregulated by UCHL1 Gene Knockdown in C2C12 Cells

**FIGURE 5:**
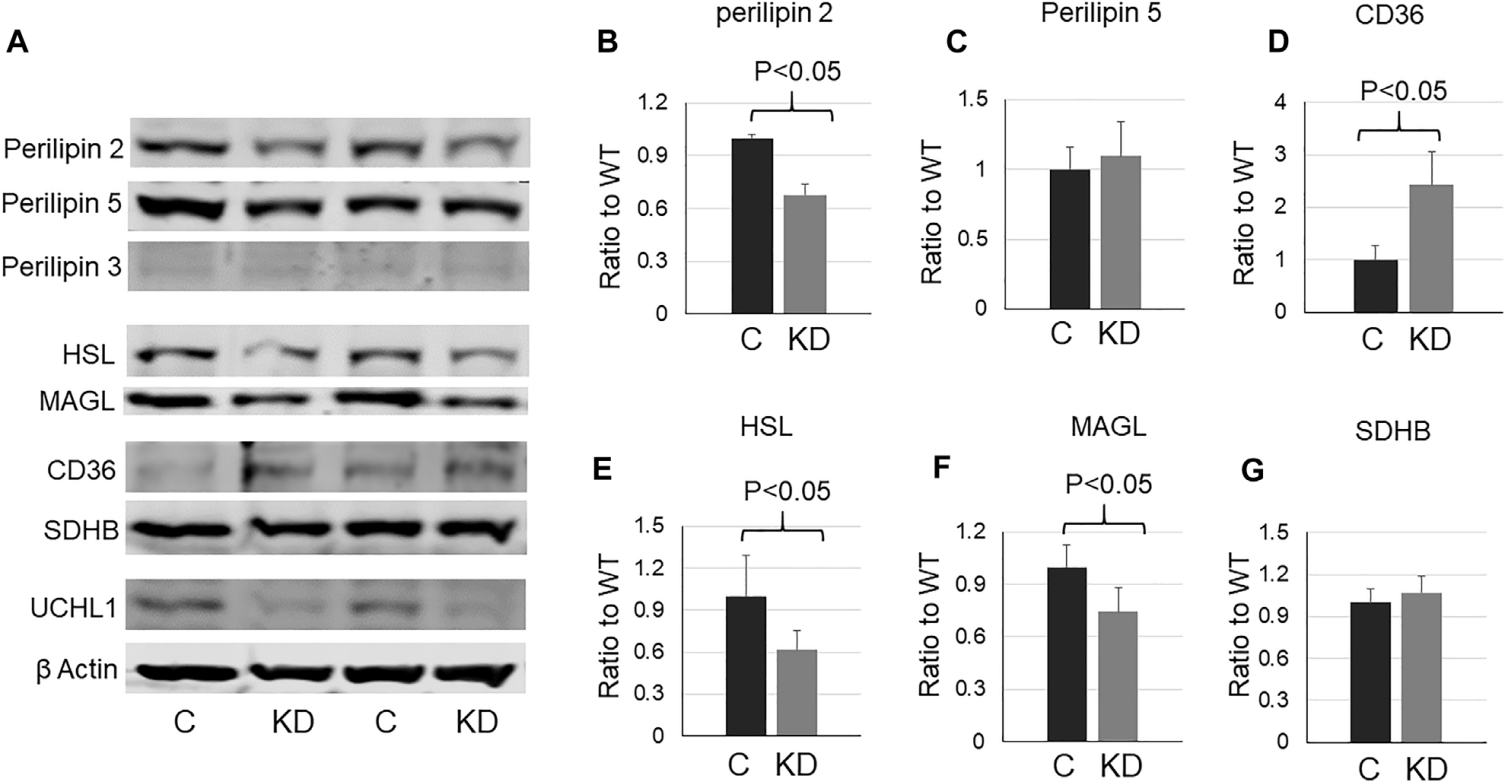
Western blot images **(A)** and quantifications **(B–G)** of cell lysates from C2C12 myotubes with control or UCHL1 siRNA knockdown for perilipin 2 **(B)**, perilipin 5 **(C)**, CD36 **(D)**, HSL **(E)**, MAGL **(F)**, and SDHB **(G)**. n = 4 per group.

To further determine whether UCHL1 regulates perilipin 2, we used siRNA to knock down (KD) UCHL1 in differentiated C2C12 myotubes. Consistent with the animal data, UCHL1 KD significantly reduced perilipin 2 protein level (Figures 5A,B). UCHL1 KD also upregulated CD36 (Figure 5D) and downregulated lipase HSL (Figure 5E) and MAGL (Figure 5F) in C2C12 cells, potentially compensatory responses to the reduced perilipin 2 and possible low lipid content. These later changes, however, were not seen in the muscle with UCHL1 KO.

### UCHL1 smKO Improved Insulin Sensitivity

**FIGURE 6:**
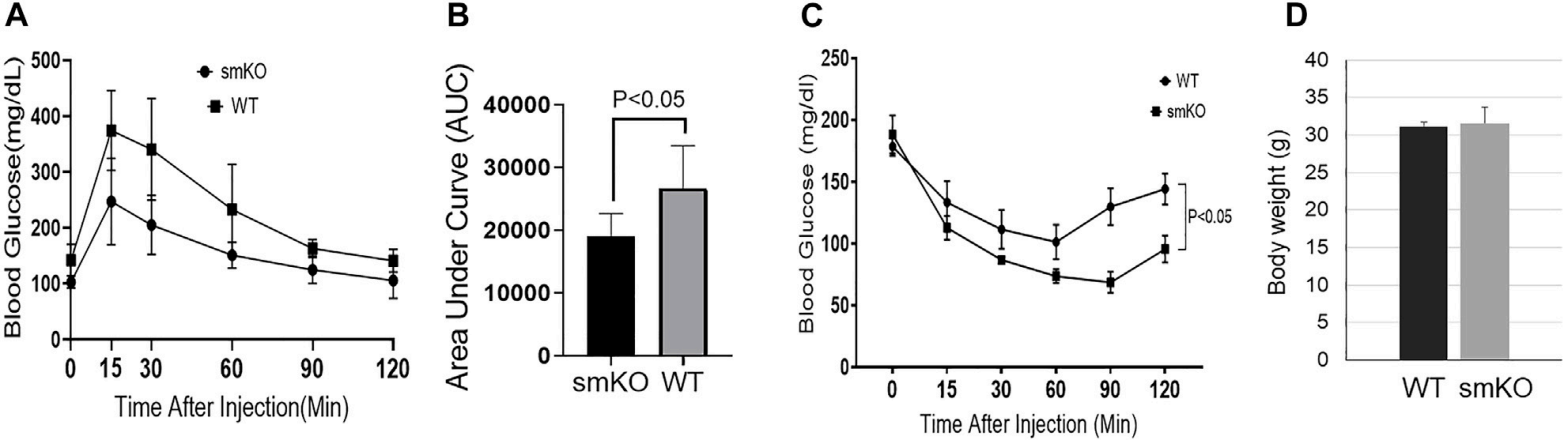
Glucose tolerance test and insulin tolerance test. **(A–B)**: GTT curve **(A)** and area under the curve **(B)**; n = 5 per group. **(C)**: ITT curve. n = 5–6 per group. **(D)**: body weight. n = 6 per group.

To test whether the reduction of intramuscular lipid affected glucose metabolism and insulin sensitivity, we conducted GTT and ITT. Mice with UCHL1 smKO have improved glucose tolerance (Figures 6A,B) as well as insulin tolerance (Figure 6C), suggesting the lower intramuscular lipid content induced by UCHL1 smKO has favorable effects on glucose metabolism and insulin sensitivity.

## Discussion

This study provides evidence for the first time showing that skeletal muscle UCHL1 is involved in regulation of intramuscular lipid content. In skeletal muscle, oxidative muscle uses both glucose and fatty acids as energy fuel, depending on the availability. When extracellular glucose levels are low, such as during fasting and exercise, the energy metabolism in oxidative muscles shifts to fatty acid oxidation. We observed that UCHL1 was highly expressed in oxidative muscle such as soleus but has very low levels in the glycolytic EDL. Moreover, skeletal muscle UCHL1 was downregulated by fasting in mice and glucose starvation in C2C12 cells, suggesting that UCHL1 may be involved in energy metabolism shift. Consistent with this, selective gene knockout of UCHL1 in skeletal muscle significantly reduced intramuscular lipid. Together, these results suggest that UCHL1 may function to facilitate lipid storage in skeletal muscle, and downregulation of skeletal muscle UCHL1 in fasting and starvation may be essential to mobilize stored lipid for lipolysis to increase free fatty acid availability for oxidation.

The level of intramuscular lipid content can be affected by many factors, including free fatty acid uptake, lipid synthesis, lipolysis, and fatty acid oxidation. Our results showed that in UCHL1 smKO skeletal muscle there were no changes with protein levels of CD36, the major fatty acid transporter (Pepino et al., 2014), ATGL, HSL, and MAGL, the major lipases (Badin et al., 2011), suggesting that the reduced intramuscular lipid by UCHL1 smKO is unlikely due to the reduced fatty acid transport and increased lipolysis. It is also unlikely due to the reduced lipid synthesis because a major lipid synthase, DGAT2, was upregulated in muscle with UCHL1 knockout, potentially a compensatory response to the low lipid levels. AMPK and Akt pathways are major signaling pathways to promote lipolysis, mitochondrial biogenesis, and fatty acid oxidation. Our data did not show any changes in total protein level and phosphorylation of AMPK and Akt, the two major pathways that regulate lipid metabolism, in UCHL1 smKO muscle. Protein levels of SDHA and PDH, two mitochondrial markers, were also unchanged in UCHL1 smKO muscle. Together, these results suggest that the reduced lipid content in UCHL1 smKO muscle may not be due to the increased fatty acid oxidation.

Intramuscular lipids are stored into lipid droplets (LDs) (Walther and Farese, 2012). LDs are active organelles that contain and store toxic lipid as energy depots (Listenberger et al., 2003). When energy fuel runs low such as during fasting or exercise, LDs can mobilize stored lipid for lipolysis to increase free fatty acids for oxidation (Rambold et al., 2015). LD membranes are embedded with different proteins, among which is the family of perilipin proteins (Kimmel and Sztalryd, 2016). Perilipin 2 (also known as adipose differentiation-related protein, ADFP) is one of the 5 proteins in the perilipin family. Perilipin 2 is highly expressed in adipose tissues and skeletal muscle in rodents and humans (Minnaard et al., 2009). This protein is not only critical for LDs membrane integrity but can also interact with major lipases such as ATGL (MacPherson et al., 2013) or be targeted by chaperone-mediated lipophagy to mobilize stored lipid for lipolysis (Kaushik and Cuervo, 2015). Our results indicate that UCHL1 knockout in mouse skeletal muscle or knockdown in C2C12 cells result in the reduction of perilipin 2 protein level. It is plausible to propose that the downregulation of perilipin 2 may be responsible for the reduction of lipid content in UCHL1 knockout muscle. Indeed, perilipin knockout resulted in reduced lipid content in myotubes (Feng et al., 2017). UCHL1 functions as a deubiquitinating enzyme, while perilipin 2 is subjected to ubiquitin-proteasome degradation (Xu et al., 2005; Masuda et al., 2006). Therefore, UCHL1 may stabilize perilipin 2 by reducing its ubiquitination and proteasome-mediated degradation; thus, UCHL1 downregulation or deletion can lead to increased degradation of perilipin 2. Fasting-induced downregulation of UCHL1 and subsequent perilipin 2 degradation may be a mechanism for increasing access to lipids in LDs for lipolysis.

Our data showed an upregulation of perilipin 3 in UCHL1 smKO muscle. This is likely a compensatory response to the downregulation of perilipin 2 and/or low muscle lipid content. The role of perilipin 3 in skeletal muscle is not clear (Morales et al., 2017). Perilipin 3 levels in muscle biopsies from healthy human subjects are positively correlated with whole-body oxidative capacity (Covington et al., 2015). Whether this correlation is associated with lipid content is unknown. In the muscle with UCHL1 smKO, the muscle lipid is low even though perilipin 3 was upregulated, suggesting the functions of perilipin 2 and perilipin 3 may not overlap, therefore, upregulation of perilipin 3 does not compensate perilipin 2 downregulation-induced reduction of muscle lipid content.

While lipids are an essential energy depot, intramuscular lipid accumulation, as seen in obesity and aging muscle, contributes to insulin resistance. We found that mice with UCHL1 smKO exhibit improved glucose tolerance and insulin tolerance, suggesting that the reduced intramuscular lipid by UCHL1 KO is protective. This is consistent with the report that perilipin 2 KO mice showed increased insulin sensitivity in obese mice (Chang et al., 2010). Therefore, the reduced perilipin 2 by UCHL1 smKO may also contribute to the increased insulin sensitivity in this study. We would like to point out that in this study, fasting plasma insulin level was not measured. Insulin sensitivity is also regulated by many factors. Therefore, the mechanisms of the enhanced glucose tolerance and insulin tolerance in UCHL1 smKO mice remain to be further investigated.

Further studies are needed to fully understand the role of skeletal muscle UCHL1 in lipid metabolism, particularly whether upregulation of UCHL1 in skeletal muscle is involved in metabolic disorders and insulin resistance. Interestingly, our previous work showed that UCHL1 skeletal muscle knockout reduced mitochondria oxidation activity (Gao et al., 2020), which seems contradictory to the present data because reduction of mitochondrial oxidation can increase lipid accumulation. One possibility for these seemingly contradictory results in the same UCHL1 smKO mouse model is that the reduced oxidative activity observed previously may be secondary to the reduced muscle lipid content, that is, reduced lipid and fatty acids lead to the reduced mitochondrial oxidation activity. This possibility certainly needs to be further verified.

## Data Availability

The original contributions presented in the study are included in the article/Supplementary Material, further inquiries can be directed to the corresponding author.

## References

[B1] Antony R. Li Y. (2020). BDNF Secretion from C2C12 Cells Is Enhanced by Methionine Restriction. Biochem. Biophysical Res. Commun. 533, 1347–1351. 10.1016/j.bbrc.2020.10.017 PMC7744331 33069357

[B2] Badin P.-M. Louche K. Mairal A. Liebisch G. Schmitz G. Rustan A. C. (2011). Altered Skeletal Muscle Lipase Expression and Activity Contribute to Insulin Resistance in Humans. Diabetes 60, 1734–1742. 10.2337/db10-1364 21498783 PMC3114384

[B3] Chang B. H.-J. Li L. Saha P. Chan L. (2010). Absence of Adipose Differentiation Related Protein Upregulates Hepatic VLDL Secretion, Relieves Hepatosteatosis, and Improves Whole Body Insulin Resistance in Leptin-Deficient Mice. J. Lipid Res. 51, 2132–2142. 10.1194/jlr.M004515 20424269 PMC2903828

[B4] Conte M. Franceschi C. Sandri M. Salvioli S. (2016). Perilipin 2 and Age-Related Metabolic Diseases: A New Perspective. Trends Endocrinol. Metab. 27, 893–903. 10.1016/j.tem.2016.09.001 27659144

[B5] Covington J. D. Noland R. C. Hebert R. C. Masinter B. S. Smith S. R. Rustan A. C. (2015). Perilipin 3 Differentially Regulates Skeletal Muscle Lipid Oxidation in Active, Sedentary, and Type 2 Diabetic Males. J. Clin. Endocrinol. Metab. 100, 3683–3692. 10.1210/JC.2014-4125 26171795 PMC4596049

[B6] Das U. N. (2001). Is Obesity an Inflammatory Condition? Nutrition 17, 953–966. 10.1016/s0899-9007(01)00672-4 11744348

[B7] Dirks M. L. Wall B. T. van de Valk B. Holloway T. M. Holloway G. P. Chabowski A. (2016). One Week of Bed Rest Leads to Substantial Muscle Atrophy and Induces Whole-Body Insulin Resistance in the Absence of Skeletal Muscle Lipid Accumulation. Diabetes 65, 2862–2875. 10.2337/db15-1661 27358494

[B8] Dominguez L. J. Barbagallo M. (2016). The Biology of the Metabolic Syndrome and Aging. Curr. Opin. Clin. Nutr. Metab. Care 19, 5–11. 10.1097/MCO.0000000000000243 26560521

[B9] Dubé J. J. Amati F. Stefanovic-Racic M. Toledo F. G. S. Sauers S. E. Goodpaster B. H. (2008). Exercise-induced Alterations in Intramyocellular Lipids and Insulin Resistance: the Athlete's Paradox Revisited. Am. J. Physiology-Endocrinology Metab. 294, E882–E888. 10.1152/ajpendo.00769.2007 PMC3804891 18319352

[B10] Dyck D. J. Peters S. J. Glatz J. Gorski J. Keizer H. Kiens B. (1997). Functional Differences in Lipid Metabolism in Resting Skeletal Muscle of Various Fiber Types. Am. J. Physiology-Endocrinology Metab. 272, E340–E351. 10.1152/ajpendo.1997.272.3.E340 9124537

[B11] Feng Y. Z. Lund J. Li Y. Knabenes I. K. Bakke S. S. Kase E. T. (2017). Loss of Perilipin 2 in Cultured Myotubes Enhances Lipolysis and Redirects the Metabolic Energy Balance from Glucose Oxidation towards Fatty Acid Oxidation. J. Lipid Res. 58, 2147–2161. 10.1194/jlr.M079764 28822960 PMC5665670

[B12] Gao H. Antony R. Srinivasan R. Wu P. Wang X. Li Y. (2020). UCHL1 Regulates Oxidative Activity in Skeletal Muscle. PLoS One 15, e0241716. 10.1371/journal.pone.0241716 33137160 PMC7605647

[B13] Gao H. Freeling J. Wu P. Liang A. P. Wang X. Li Y. (2019). UCHL1 Regulates Muscle Fibers and mTORC1 Activity in Skeletal Muscle. Life Sci. 233, 116699. 10.1016/j.lfs.2019.116699 31356902 PMC6718320

[B14] Goodpaster B. H. He J. Watkins S. Kelley D. E. (2001). Skeletal Muscle Lipid Content and Insulin Resistance: Evidence for a Paradox in Endurance-Trained Athletes. J. Clin. Endocrinol. Metab. 86, 5755–5761. 10.1210/jcem.86.12.8075 11739435

[B15] Hirabara S. M. Silveira L. R. Abdulkader F. Carvalho C. R. O. Procopio J. Curi R. (2007). Time-dependent Effects of Fatty Acids on Skeletal Muscle Metabolism. J. Cel. Physiol. 210, 7–15. 10.1002/jcp.20811 17013887

[B16] Houten S. M. Violante S. Ventura F. V. Wanders R. J. A. (2016). The Biochemistry and Physiology of Mitochondrial Fatty Acid β-Oxidation and its Genetic Disorders. Annu. Rev. Physiol. 78, 23–44. 10.1146/annurev-physiol-021115-105045 26474213

[B17] Kaushik S. Cuervo A. M. (2015). Degradation of Lipid Droplet-Associated Proteins by Chaperone-Mediated Autophagy Facilitates Lipolysis. Nat. Cel Biol 17, 759–770. 10.1038/ncb3166 PMC4449813 25961502

[B18] Kelley D. E. (2005). Skeletal Muscle Fat Oxidation: Timing and Flexibility Are Everything. J. Clin. Invest. 115, 1699–1702. 10.1172/JCI25758 16007246 PMC1159159

[B19] Kimmel A. R. Brasaemle D. L. McAndrews-Hill M. Sztalryd C. Londos C. (2010). Adoption of PERILIPIN as a Unifying Nomenclature for the Mammalian PAT-Family of Intracellular Lipid Storage Droplet Proteins. J. Lipid Res. 51, 468–471. 10.1194/jlr.R000034 19638644 PMC2817576

[B20] Kimmel A. R. Sztalryd C. (2016). The Perilipins: Major Cytosolic Lipid Droplet-Associated Proteins and Their Roles in Cellular Lipid Storage, Mobilization, and Systemic Homeostasis. Annu. Rev. Nutr. 36, 471–509. 10.1146/annurev-nutr-071813-105410 27431369

[B21] Koves T. R. Ussher J. R. Noland R. C. Slentz D. Mosedale M. Ilkayeva O. (2008). Mitochondrial Overload and Incomplete Fatty Acid Oxidation Contribute to Skeletal Muscle Insulin Resistance. Cel Metab. 7, 45–56. 10.1016/j.cmet.2007.10.013 18177724

[B22] Kumar A. Accorsi A. Rhee Y. Girgenrath M. (2015). Do's and Don'ts in the Preparation of Muscle Cryosections for Histological Analysis. JoVE 99, e52793. 10.3791/52793 PMC4542757 26066009

[B23] Lalia A. Z. Dasari S. Johnson M. L. Robinson M. M. Konopka A. R. Distelmaier K. (2016). Predictors of Whole-Body Insulin Sensitivity across Ages and Adiposity in Adult Humans. J. Clin. Endocrinol. Metab. 101, 626–634. 10.1210/jc.2015-2892 26709968 PMC4880121

[B24] Listenberger L. L. Han X. Lewis S. E. Cases S. Farese R. V. Ory D. S. (2003). Triglyceride Accumulation Protects against Fatty Acid-Induced Lipotoxicity. Proc. Natl. Acad. Sci. U.S.A. 100, 3077–3082. 10.1073/pnas.0630588100 12629214 PMC152249

[B25] Lundsgaard A.-M. Fritzen A. M. Kiens B. (2018). Molecular Regulation of Fatty Acid Oxidation in Skeletal Muscle during Aerobic Exercise. Trends Endocrinol. Metab. 29, 18–30. 10.1016/j.tem.2017.10.011 29221849

[B26] MacPherson R. E. K. Ramos S. V. Vandenboom R. Roy B. D. Peters S. J. (2013). Skeletal Muscle PLIN Proteins, ATGL and CGI-58, Interactions at Rest and Following Stimulated Contraction. Am. J. Physiology-Regulatory, Integr. Comp. Physiol. 304, R644–R650. 10.1152/ajpregu.00418.2012 PMC3627954 23408028

[B27] Masuda Y. Itabe H. Odaki M. Hama K. Fujimoto Y. Mori M. (2006). ADRP/adipophilin Is Degraded through the Proteasome-dependent Pathway during Regression of Lipid-Storing Cells. J. Lipid Res. 47, 87–98. 10.1194/jlr.M500170-JLR200 16230742

[B28] Minnaard R. Schrauwen P. Schaart G. Jorgensen J. A. Lenaers E. Mensink M. (2009). Adipocyte Differentiation-Related Protein and OXPAT in Rat and Human Skeletal Muscle: Involvement in Lipid Accumulation and Type 2 Diabetes Mellitus. J. Clin. Endocrinol. Metab. 94, 4077–4085. 10.1210/jc.2009-0352 19602560

[B29] Morales P. E. Bucarey J. L. Espinosa A. (2017). Muscle Lipid Metabolism: Role of Lipid Droplets and Perilipins. J. Diabetes Res. 2017, 1–10. 10.1155/2017/1789395 PMC5476901 28676863

[B30] Ogasawara Y. Tsuji T. Fujimoto T. (2020). Multifarious Roles of Lipid Droplets in Autophagy - Target, Product, and what Else? Semin. Cel Dev. Biol. 108, 47–54. 10.1016/j.semcdb.2020.02.013 32169402

[B31] Olzmann J. A. Carvalho P. (2019). Dynamics and Functions of Lipid Droplets. Nat. Rev. Mol. Cel Biol 20, 137–155. 10.1038/s41580-018-0085-z PMC6746329 30523332

[B32] Pepino M. Y. Kuda O. Samovski D. Abumrad N. A. (2014). Structure-function of CD36 and Importance of Fatty Acid Signal Transduction in Fat Metabolism. Annu. Rev. Nutr. 34, 281–303. 10.1146/annurev-nutr-071812-161220 24850384 PMC4329921

[B33] Qiu B. Simon M. (2016). BODIPY 493/503 Staining of Neutral Lipid Droplets for Microscopy and Quantification by Flow Cytometry. Bio-protocol 6, e1912. 10.21769/BioProtoc.1912 28573161 PMC5448404

[B34] Rambold A. S. Cohen S. Lippincott-Schwartz J. (2015). Fatty Acid Trafficking in Starved Cells: Regulation by Lipid Droplet Lipolysis, Autophagy, and Mitochondrial Fusion Dynamics. Dev. Cel 32, 678–692. 10.1016/j.devcel.2015.01.029 PMC4375018 25752962

[B35] Reidy P. T. McKenzie A. I. Mahmassani Z. Morrow V. R. Yonemura N. M. Hopkins P. N. (2018). Skeletal Muscle Ceramides and Relationship with Insulin Sensitivity after 2 Weeks of Simulated Sedentary Behaviour and Recovery in Healthy Older Adults. J. Physiol. 596, 5217–5236. 10.1113/JP276798 30194727 PMC6209761

[B36] Samuel V. T. Petersen K. F. Shulman G. I. (2010). Lipid-induced Insulin Resistance: Unravelling the Mechanism. The Lancet 375, 2267–2277. 10.1016/S0140-6736(10)60408-4 PMC2995547 20609972

[B37] Schiaffino S. Reggiani C. (2011). Fiber Types in Mammalian Skeletal Muscles. Physiol. Rev. 91, 1447–1531. 10.1152/physrev.00031.2010 22013216

[B38] Silveira L. R. Fiamoncini J. Hirabara S. M. Procópio J. Cambiaghi T. D. Pinheiro C. H. J. (2008). Updating the Effects of Fatty Acids on Skeletal Muscle. J. Cel. Physiol. 217, 1–12. 10.1002/jcp.21514 18543263

[B39] Spangenburg E. E. Pratt S. J. P. Wohlers L. M. Lovering R. M. (20112011). Use of BODIPY (493/503) to Visualize Intramuscular Lipid Droplets in Skeletal Muscle. J. Biomed. Biotechnol. 2011, 1–8. 10.1155/2011/598358 PMC3180081 21960738

[B40] Stein T. P. Wade C. E. (2005). Metabolic Consequences of Muscle Disuse Atrophy. J. Nutr. 135, 1824S–1828S. 10.1093/jn/135.7.1824S 15987873

[B41] Storlien L. Oakes N. D. Kelley D. E. (2004). Metabolic Flexibility. Proc. Nutr. Soc. 63, 363–368. 10.1079/PNS2004349 15294056

[B42] Turcotte L. P. Fisher J. S. (2008). Skeletal Muscle Insulin Resistance: Roles of Fatty Acid Metabolism and Exercise. Phys. Ther. 88, 1279–1296. 10.2522/ptj.20080018 18801860 PMC2579902

[B43] Turner N. Cooney G. J. Kraegen E. W. Bruce C. R. (2014). Fatty Acid Metabolism, Energy Expenditure and Insulin Resistance in Muscle. J. Endocrinol. 220, T61–T79. 10.1530/JOE-13-0397 24323910

[B44] Walther T. C. Farese R. V. Jr. (2012). Lipid Droplets and Cellular Lipid Metabolism. Annu. Rev. Biochem. 81, 687–714. 10.1146/annurev-biochem-061009-102430 22524315 PMC3767414

[B45] Wang C.-W. (2016). Lipid Droplets, Lipophagy, and beyond. Biochim. Biophys. Acta (Bba) - Mol. Cel Biol. Lipids 1861, 793–805. 10.1016/j.bbalip.2015.12.010 26713677

[B46] Xu G. Sztalryd C. Lu X. Tansey J. T. Gan J. Dorward H. (2005). Post-translational Regulation of Adipose Differentiation-Related Protein by the Ubiquitin/proteasome Pathway. J. Biol. Chem. 280, 42841–42847. 10.1074/jbc.M506569200 16115879

