# Supplementary material for: UCHL1 Regulates Lipid and Perilipin 2 Level in Skeletal Muscle
Source: Front Physiol. 2022 Apr 7;13:855193. doi: 10.3389/fphys.2022.855193 (PMC9021748; doi:10.3389/fphys.2022.855193)

Figure 1A GAPDH

SOL EDL SOL EDL SOL EDL SOL EDL

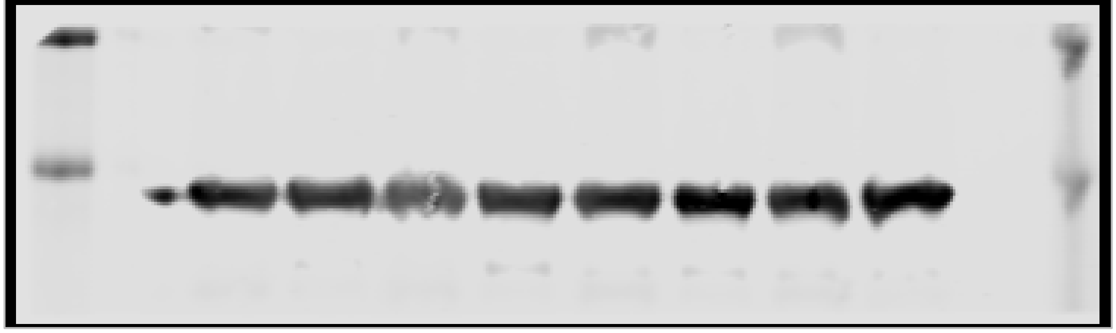

Figure 1A UCHL1

SOL EDL SOL EDL SOL EDL SOL EDL

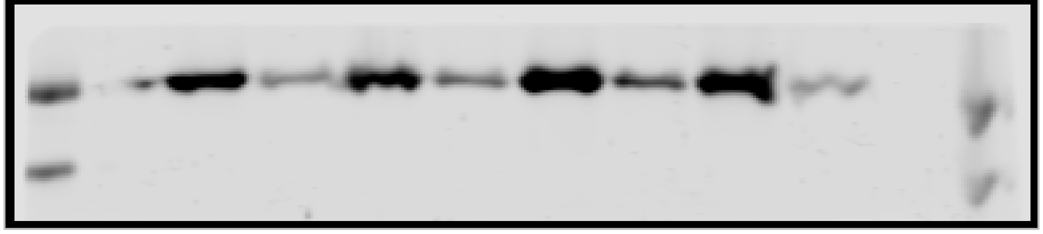

Figure 1B GAPDH and UCHL1

fed fasted fed fasted fed fasted

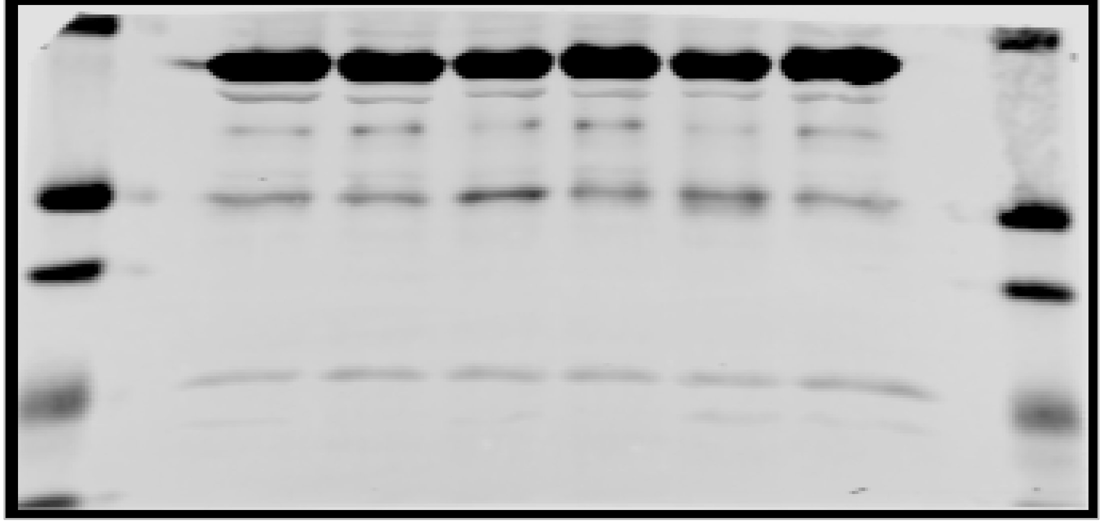

Figure 1C Actin

CM ICM NG CM ICM NG CM ICM NG

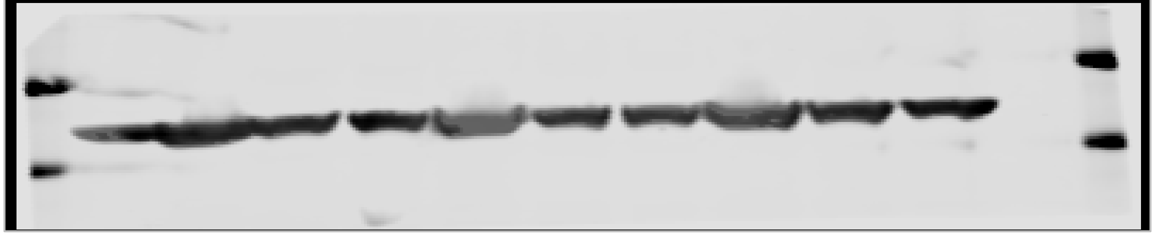

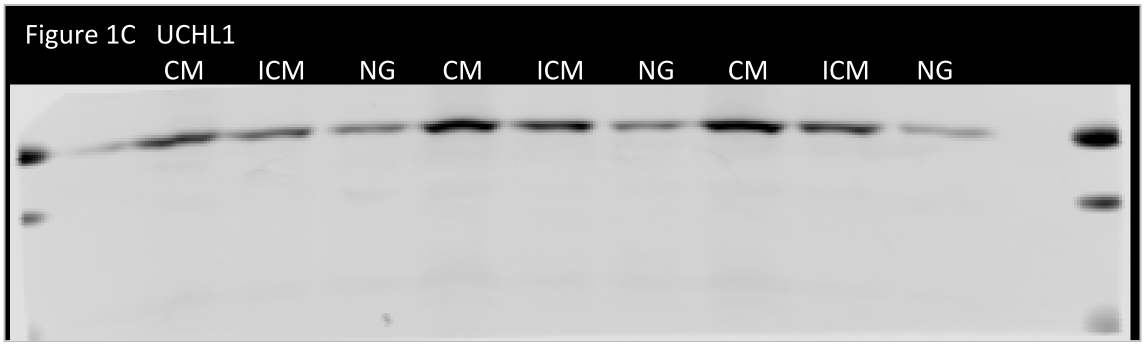
Figure 1C UCHL1
CM ICM NG CM ICM NG CM ICM NG

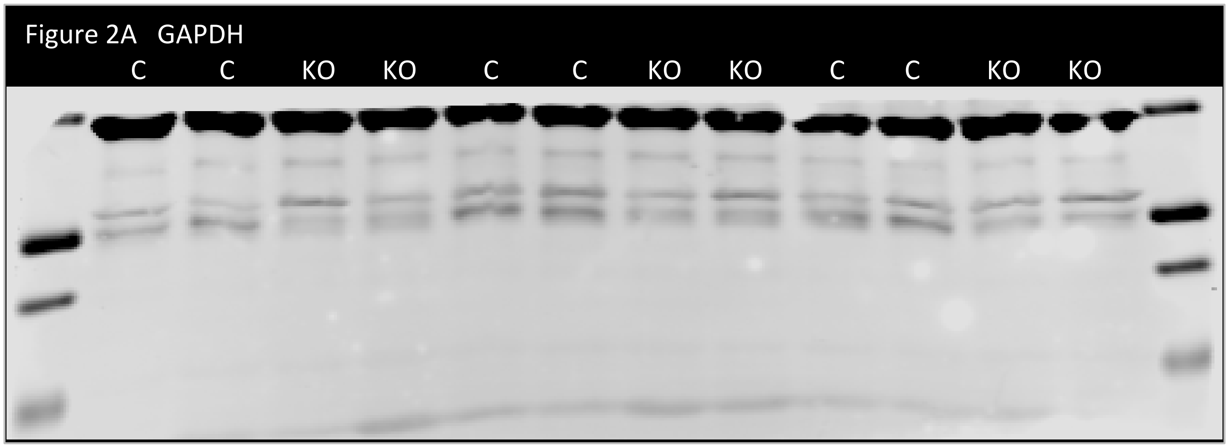
Figure 2A GAPDH
C C KO KO C C KO KO C C KO KO

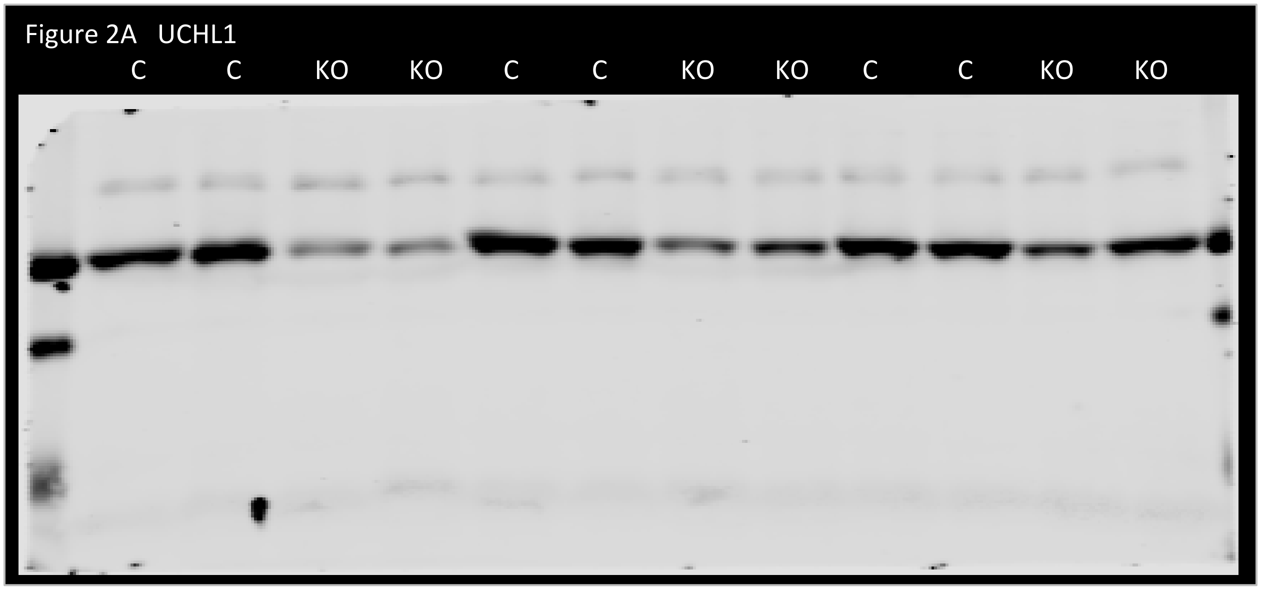
Figure 2A UCHL1
C C KO KO C C KO KO C C KO KO

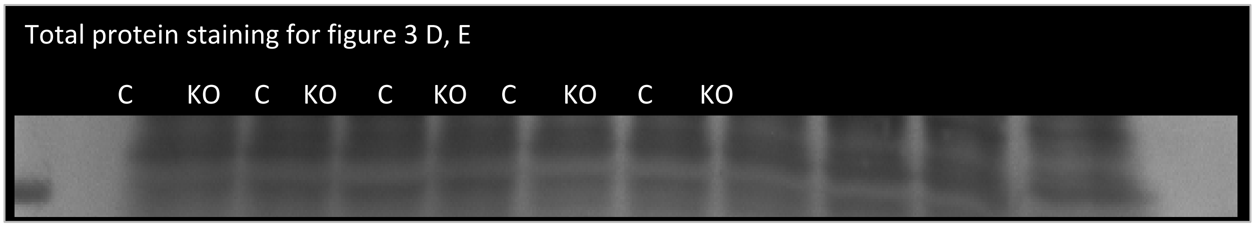
Total protein staining for figure 3 D, E
C KO C KO C KO C KO C KO

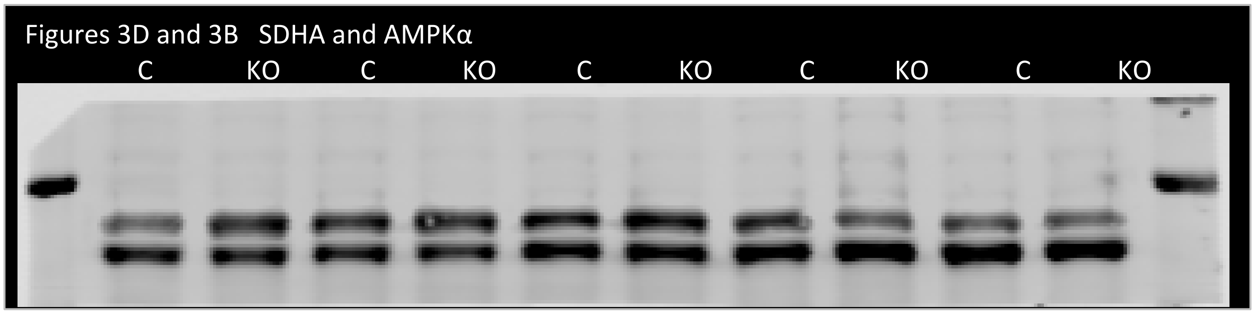
Figures 3D and 3B SDHA and AMPKα
C KO C KO C KO C KO C KO

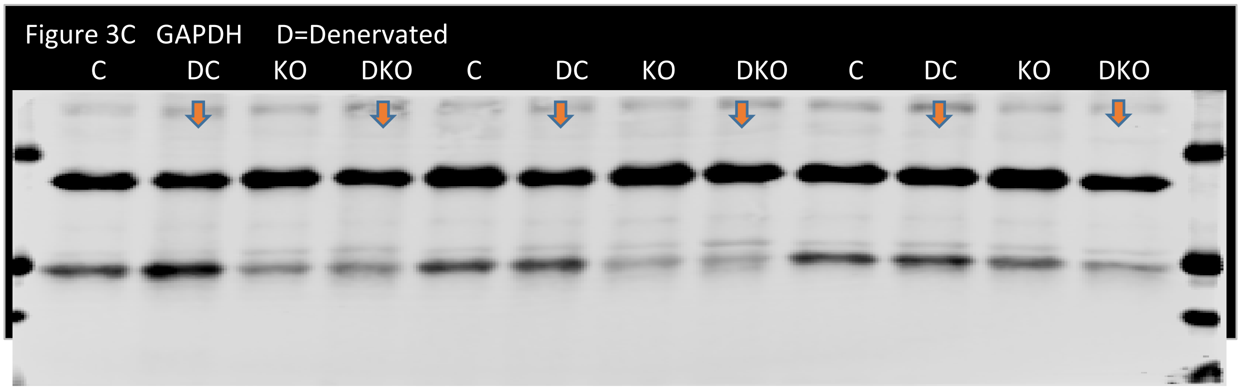
Figure 3C GAPDH D=Denervated
C DC KO DKO C DC KO DKO C DC KO DKO

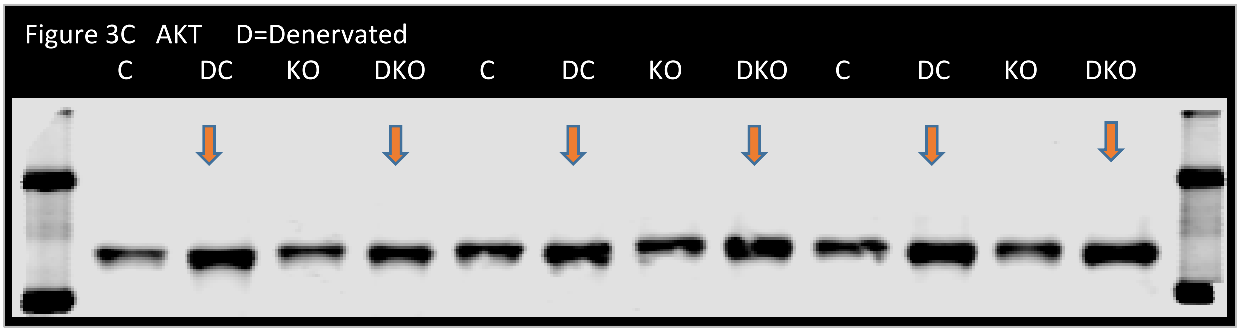
Figure 3C AKT D=Denervated
C DC KO DKO C DC KO DKO C DC KO DKO

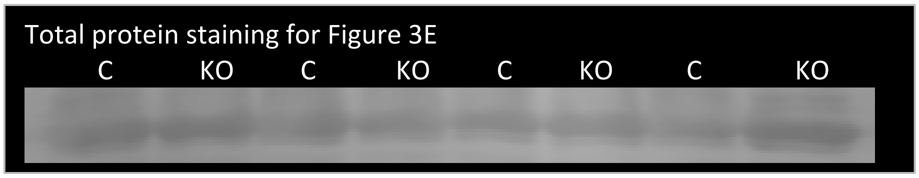
Total protein staining for Figure 3E
C KO C KO C KO C KO

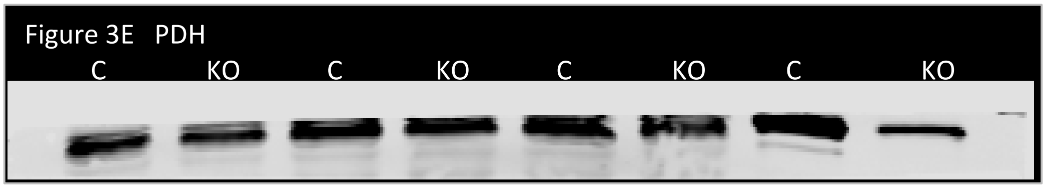
Figure 3E PDH
C KO C KO C KO C KO

Total protein staining Figure 4 B, H, and I

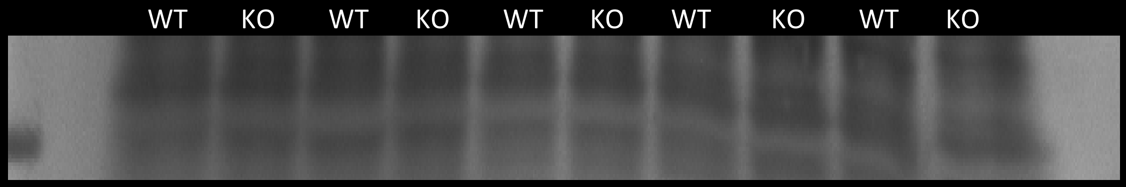

Figure 4I UCHL1

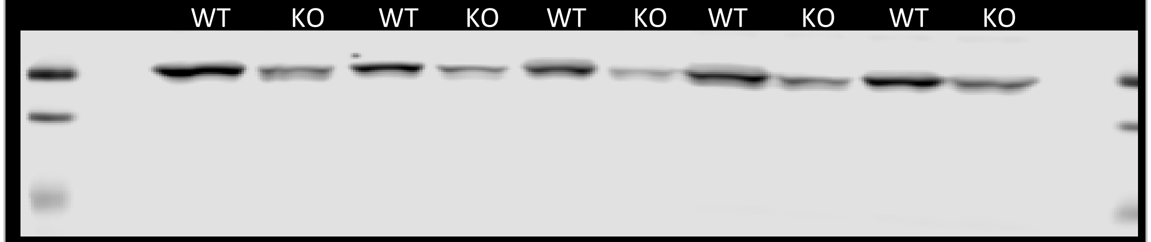

Figure 4H Perilipin 2

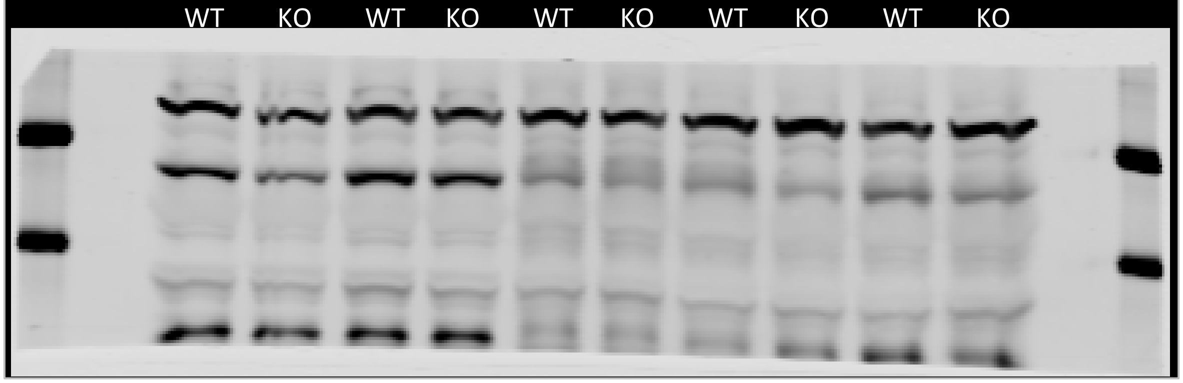

Figure 4B ATGL

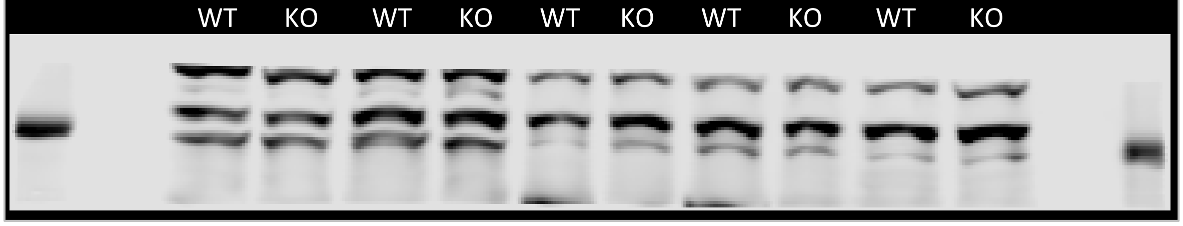

Total protein staining for Figure 4C , D, E, F, and G

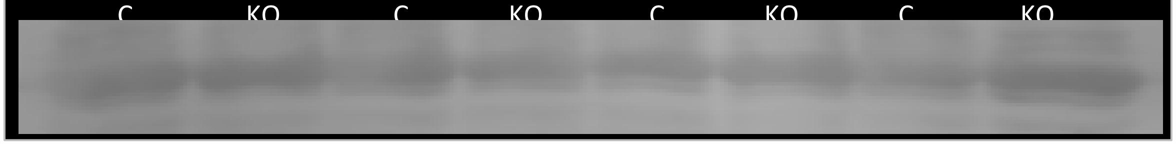

Figure 4C HSL

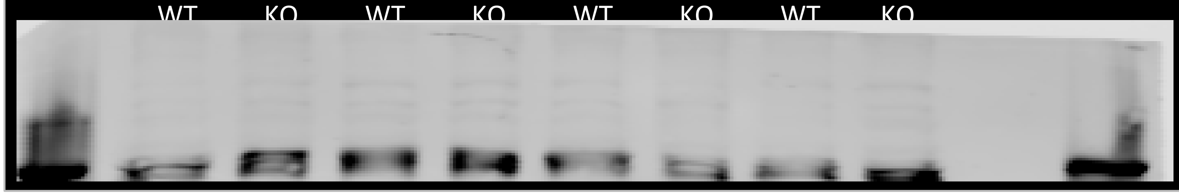

Figure 4D MAGL

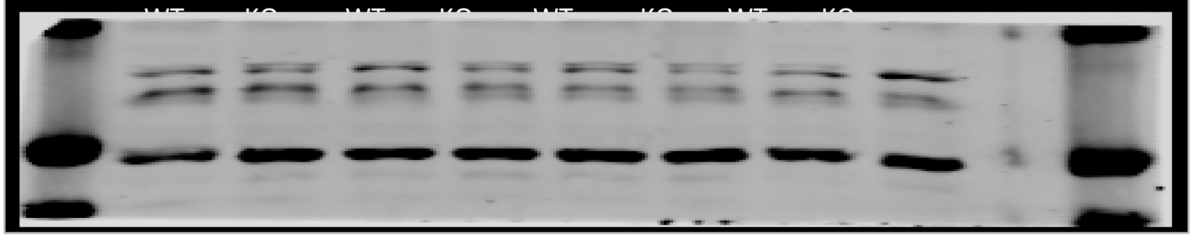

Figure 4E CD36

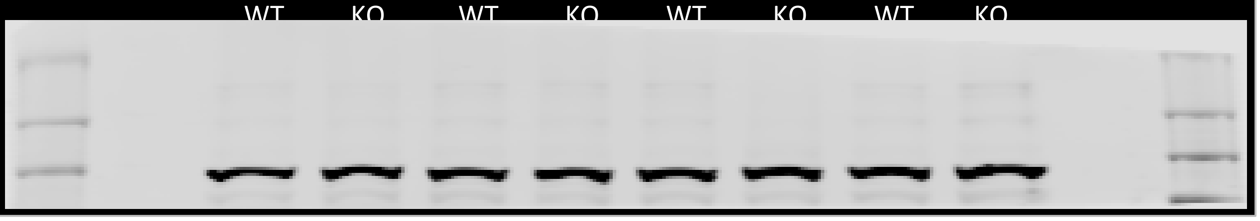

Figure 4G Perilipin 3

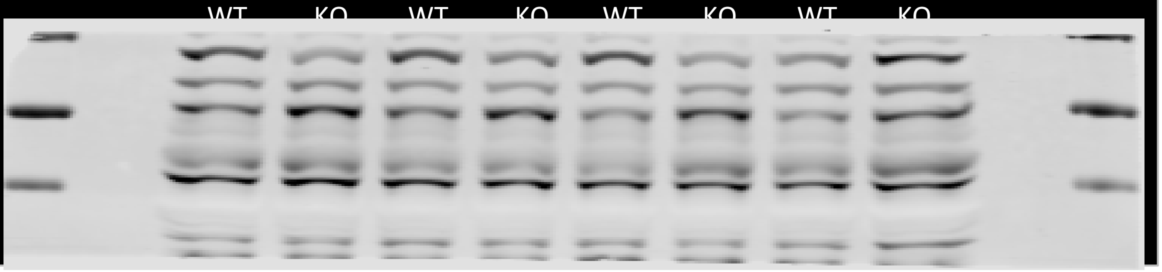

Figure 4F DGAT2

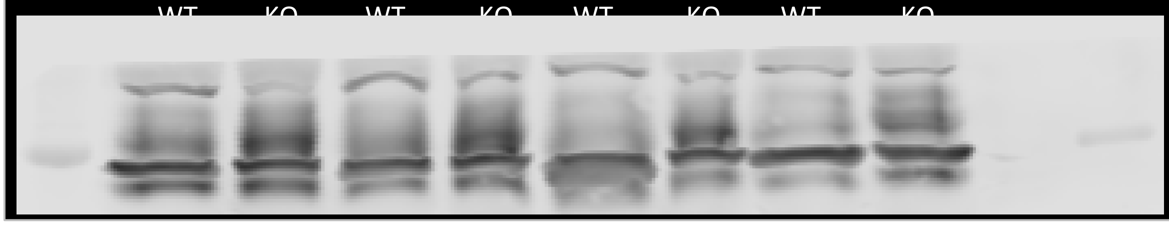

Figure 5 Actin

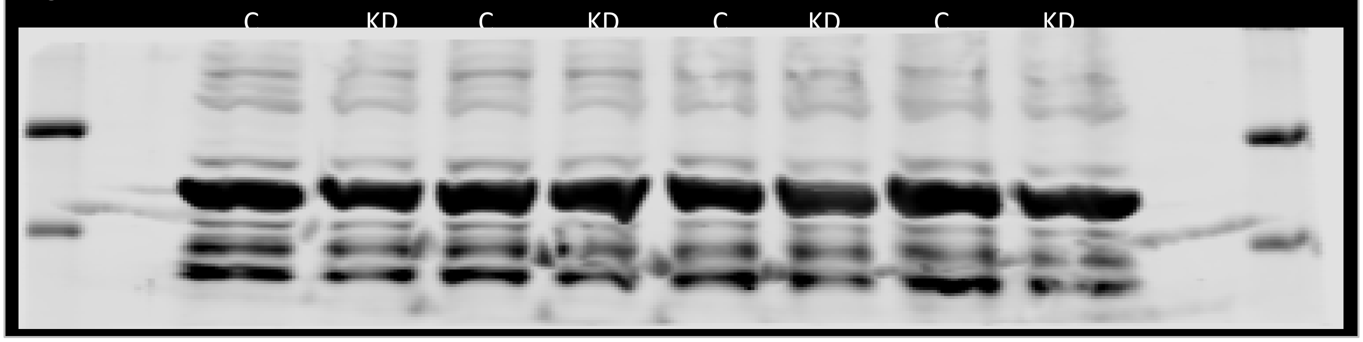

Figure 5 UCHL1

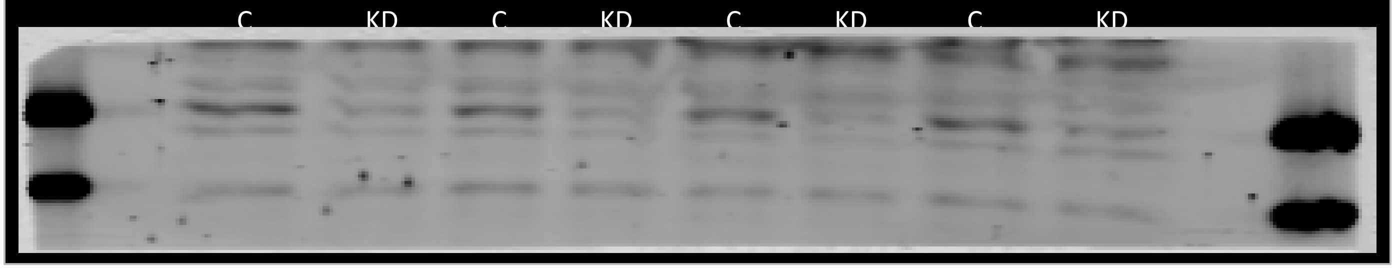

Figure 5B Perilipin 2

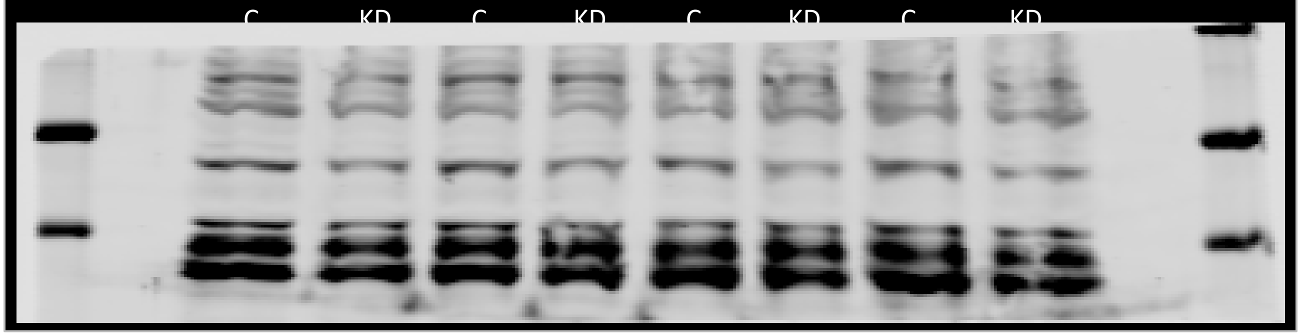

Figure 5E HSL

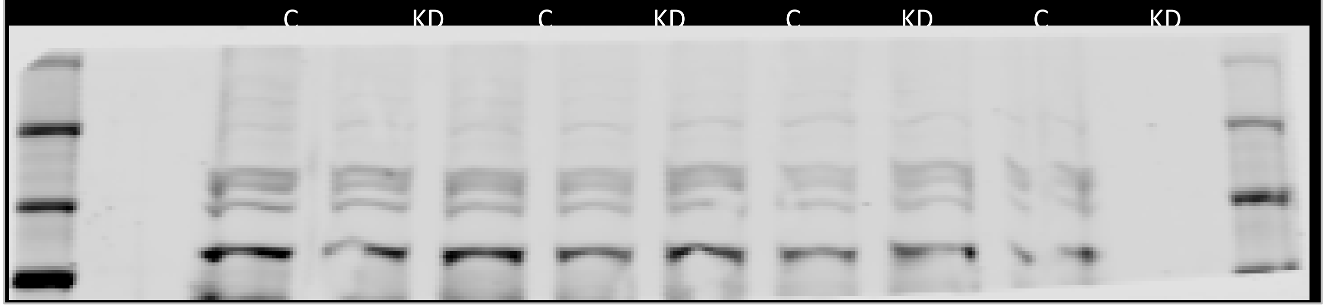

Figure 5G SDHB

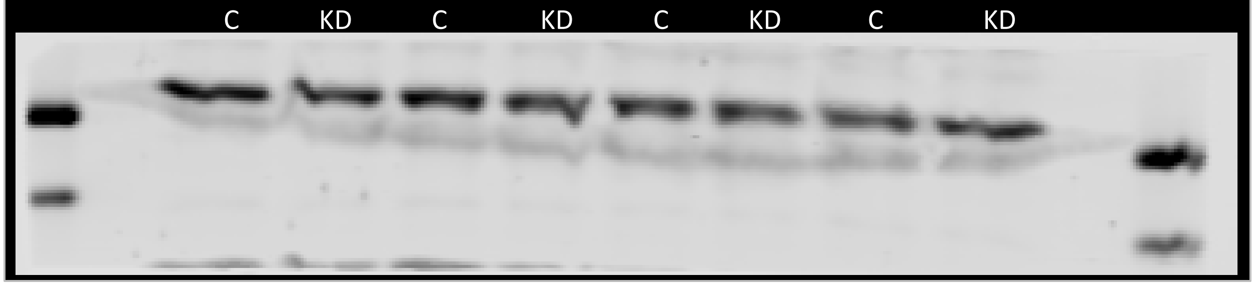

Figure 5F MAGL

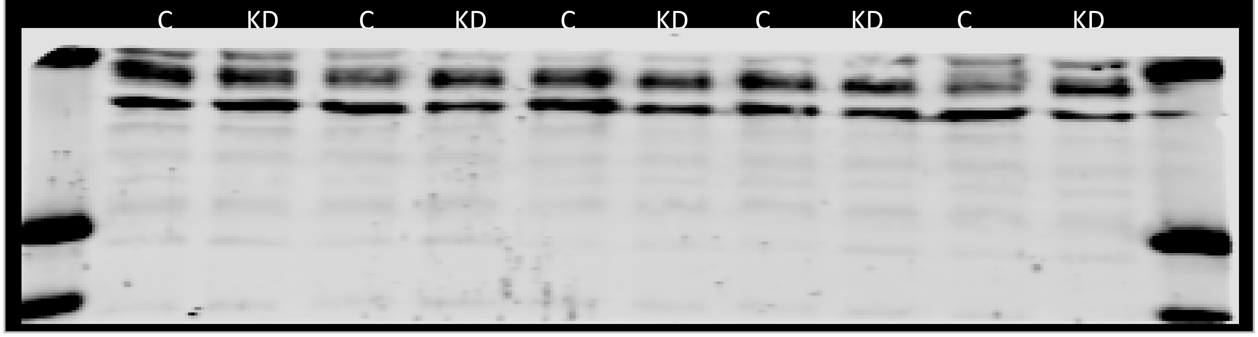

Figure 5D CD36

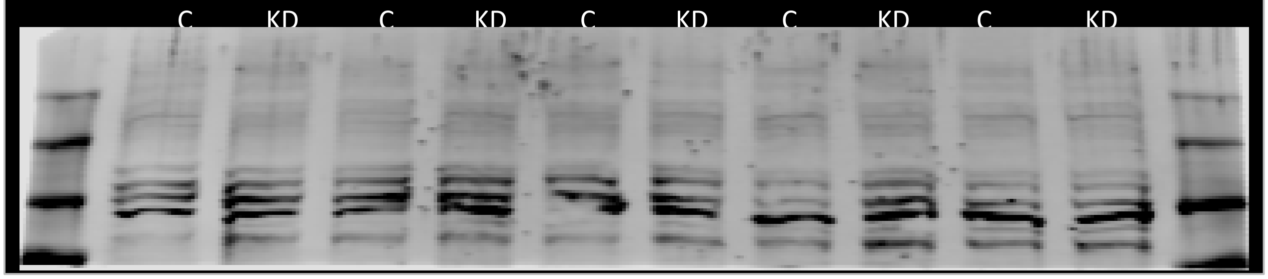

Figure 5C Perilipin 5

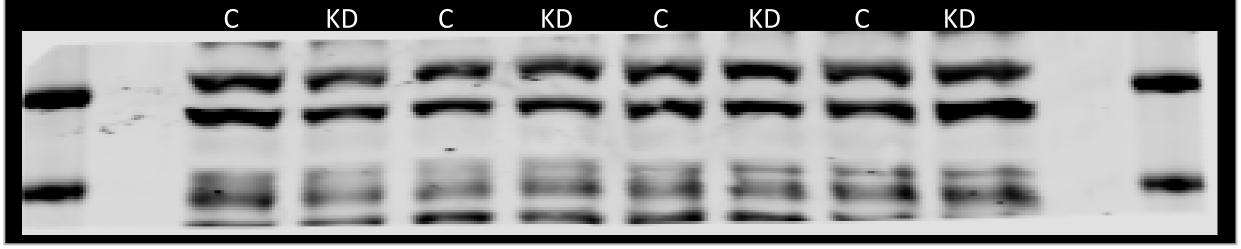

Figure 5 Perilipin 3

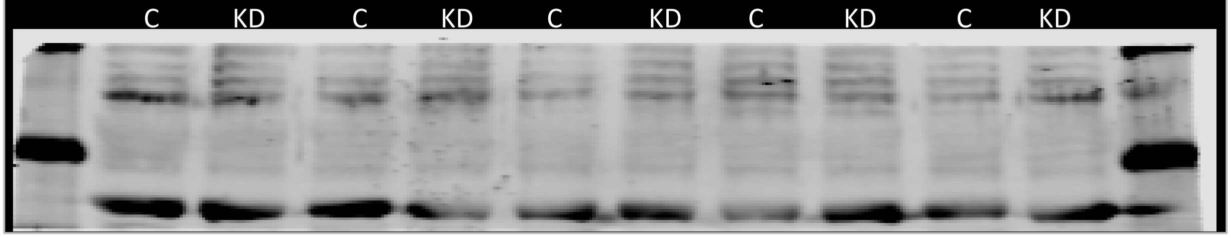

Supplement: Supplementary file 1 [file Image1.pdf]
